# High-density lipoprotein (HDL) particle size and concentration changes in septic shock patients

**DOI:** 10.1186/s13613-019-0541-8

**Published:** 2019-06-13

**Authors:** Sébastien Tanaka, Dévy Diallo, Sandrine Delbosc, Claire Genève, Nathalie Zappella, Jennyfer Yong-Sang, Jessica Patche, Anatole Harrois, Sophie Hamada, Erick Denamur, Philippe Montravers, Jacques Duranteau, Olivier Meilhac

**Affiliations:** 1 INSERM, UMR 1188 Diabète athérothombose Réunion Océan Indien (DéTROI), Université de La Réunion, 2 Rue Maxime Rivière, 97491 Sainte Clotilde, La Réunion, France; 2 AP-HP, Service d’Anesthésie-Réanimation, CHU Bichat-Claude Bernard, 46 Rue Henri Huchard, 75018 Paris, France; 3 Inserm U1148, Laboratory for Vascular, Translational Science Bichat Hospital, 46 Rue Henri Huchard, 75018 Paris, France; 4 AP-HP, Service d’Anesthésie-Réanimation, Hôpitaux Universitaires Paris-Sud, Université Paris-Sud, Hôpital de Bicêtre, 78 Rue du Général Leclerc, 94270 Le Kremlin-Bicêtre, France; 5 Laboratoire d’étude de la Microcirculation, «Bio-CANVAS: Biomarkers in CardioNeuroVascular DISEASES» UMRS 942, Paris, France; 6 UMR1137 IAME, Inserm, Laboratoire de Génétique Moléculaire, Université Paris Diderot and AP-HP, Hôpital Bichat, Paris, France; 7 Inserm UMR1152, Physiopathologie et Epidémiologie des Maladies Respiratoires, Paris, France; 8 CHU de La Réunion, Saint-Denis, France; 9 INSERM U1188 Diabète athérothrombose Thérapies Réunion Océan Indien (DéTROI), Université de La Réunion au CYROI, 2, Rue Maxime Rivière, 97490 Sainte Clotilde, La Réunion, France

**Keywords:** Inflammation, Intensive care unit, Lipoprint, Lipid profile, Sepsis, High-density lipoproteins, Size

## Abstract

**Background:**

Sepsis is associated with systemic inflammation that may impact lipoprotein function. In particular, high-density lipoproteins (HDLs) that display pleiotropic protective roles may be dysfunctional in septic conditions. The aim of this study was to evaluate the HDL profile and the inflammatory context in septic shock patients admitted to our intensive care unit (ICU).

**Methods:**

In this study, 20 septic shock patients and 20 controls (ICU patients without septic shock) were included. Plasma samples were collected on days 1, 2 and 7. Total cholesterol and lipoprotein concentrations were determined. HDL profiles were obtained using the Lipoprint^®^ System (non-denaturing electrophoresis). Quantification of pro-inflammatory cytokines (interleukin 1b, 6 and 8), cell-free DNA and lipopolysaccharide-binding protein was also performed.

**Results:**

HDL concentration was statistically lower in septic shock patients than in controls. At days 1 and 2, septic patients had significantly more large-sized HDL than control patients. Patients recovered a normal lipid profile at day 7.

**Conclusions:**

Our results emphasize that HDL levels are dramatically decreased in the acute phase of septic shock and that there is a shift toward large HDL particles, which may reflect a major dysfunction of these lipoproteins. Further mechanistic studies are required to explore this shift observed during sepsis.

**Electronic supplementary material:**

The online version of this article (10.1186/s13613-019-0541-8) contains supplementary material, which is available to authorized users.

## Background

Sepsis is still the major cause of mortality in critically ill patients [1–3]. Inflammation associated with sepsis is characterized by increased levels of circulating biomarkers including chemokines, cytokines, coagulation factors, etc., that reflect and participate in organ dysfunction [4, 5]. Previous studies have suggested that during sepsis, multiple organ dysfunctions are consecutive to endothelial alterations leading to platelet and leukocyte activation and/or coagulation pathway perturbations [6, 7]. High-density lipoproteins (HDLs), in addition to their function of reverse cholesterol transport [8, 9], display pleiotropic properties such as the induction of nitric oxide (NO) production by endothelial cells [10], inhibition of platelet activation [11] or the capacity to neutralize lipopolysaccharides (LPS) [12, 13]. HDLs have also been reported to modulate neutrophil activation as the endothelial response to pro-inflammatory cytokines such as TNF-alpha [14]. HDLs may bind to enzymes such as paraoxonase (PON1) or platelet-activating factor acetylhydrolase (PAF-AH) which display antioxidant and endothelium protective properties [13]. Both PON1 and PAF-AH have been shown to limit lipid oxidation [13, 15].

In the cardiovascular field, both HDL cholesterol (HDL-C) concentration and HDL particle size were independently associated with cardiovascular risk [16]. In particular, large HDL particles appear to be protective in coronary artery disease [17, 18]. To our knowledge, HDL size has never been explored in septic patients.

Clinical studies have emphasized that HDL-C rapidly decreased during sepsis [19, 20] and that low levels of HDL-C are associated with increased mortality and adverse clinical outcomes during sepsis [21–23]. In a previous study, we have compared HDL profiles between septic and trauma patients [20]. Although inflammation was exacerbated in these two entities, HDL-C levels were low in septic patients, whereas their concentration was not altered in cases of trauma. The anti-inflammatory properties of HDLs and their capacity to limit endothelial activation are thus decreased in septic conditions. We hypothesized that in addition to reduced HDL concentration, their size may be modified in septic conditions.

## Materials and methods

The aim of the present study was to characterize HDL particles concentration and size in septic shock patients hospitalized in our intensive care unit.

### Patients

The study was performed in a surgical intensive care unit (ICU) of a 1000-bed tertiary referral university hospital. All adult patients admitted to the ICU were enrolled in this prospective observational study when they fulfilled the criteria for septic shock according to the Surviving Sepsis Campaign International Guidelines. This study was approved by our local ethics committee (Comité de Protection des Personnes de l’Université Paris VII no SC 13-026), which waived the need for written informed consent because of the observational nature of the study. During this period, all non-septic patients hospitalized in the ICU were prospectively included in a control group.

No differences for age or sex were observed between the two groups. The main ICU admission diagnoses were severe trauma, acute severe bleeding, acute brain injury or intracerebral hemorrhage. Immuno-compromised patients (AIDS, neutropenia of < 1000 cells/mL or transplant surgery) and patients with liver cirrhosis were excluded from the study.

According to our local procedures, early enteral feeding was always preferred. If the enteral route was not possible, a parenteral nutrition was administrated and changed for enteral nutrition as soon as possible. Some patients received both enteral and parenteral nutrition.

### Data collection

Demographic data were collected at the patient’s admission (age, sex, SAPSII, SOFA score, weight, previous medical history and especially dyslipidemia and ICU admission diagnosis). Hyperlipidemia is defined as an elevation of the fasting total cholesterol concentration, which may or may not be associated with increased triglyceride concentration. Statin treatment is recommended according to international guidelines [24]. Blood was sampled at days 1, 2 and 7 after admission for determination of total cholesterol, HDL-C, triglycerides, LDL cholesterol (LDL-C), protein concentrations and leukocyte count.

### Determination of HDL profiles by the Quantimetrix Lipoprint^®^ HDL system

HDL size profiles were obtained using Lipoprint technology (Eurobio, France). The separation of HDL subfractions in plasma was achieved using this non-denaturing electrophoresis system. Precast high-resolution 8.5% polyacrylamide gel tubes were used. Briefly, 40 µl of sample was mixed with 300 µl of loading gel. This mixture was added to the top of the gel tubes, and a photo polymerization was carried out at room temperature for 30 min. Then, the gel tubes containing samples were electrophoresed for 54 min with 3 mA/gel. When the electrophoresis was finished, the gel tubes were left to rest for 30 min before scanning. This incubation at ambient temperature was added to the protocol to increase the uniformity of the bands. After scanning with a ScanMaker 8700 digital scanner (Mikrotek Co, USA), analysis with lipoware software was performed and the different bands (fractions of lipoproteins) were identified by their mobility (Rf) using a starting reference point and a leading point.

### Apolipoprotein A-I ELISA

The plasma apo A-I concentration was determined using a commercial kit (Mabtech, Sweden). The whole procedure was performed at room temperature. A high protein-binding ELISA plate was coated overnight with mAb HDL diluted to 2 µg/ml in PBS, pH 7.4 (100 µl in each well). The plate was washed twice with PBS, and blocked by adding 150 µl (by well) of PBS containing 0.05% Tween 20 and 0.1% BSA (incubation buffer) and leaving for 1 h. Five washes were then performed with PBS containing 0.05% Tween 20. 100 µl per well of diluted patient’s plasma (1:100,000) and standards (dilution range from 50 to 0 ng/ml) was incubated for 2 h. A washing step similar to the second one was then performed. Then, 100 µl/well of mAb HDL linked to biotin at 0.5 µl/ml was added and incubated for 1 h after which another washing step was performed. Streptavidin-HRP diluted at 1:1000 in incubation buffer was then added (100 µl/well). After 1 h of incubation followed by fives washes, 100 µl of substrate were added to each well, and after 5 min, the colorimetric reaction was stopped by adding 50 µl of H2SO4 1 M. The optical density was measured at 405 nm.

### LPS-binding protein (LBP) ELISA

LBP concentrations were measured using a commercial ELISA kit (Cell Sciences Inc), samples were diluted 1:1000, and the assay was carried out according to the manufacturer’s protocol, with a standard curve ranging from 5 to 50 μg/mL.

### Cell-free DNA quantification

Determination of the quantity of free DNA in plasma of patients was achieved using Quantit™ Picogreen dsDNA Reagent (Life technologies, France), as previously described [25].

### Cytokines ELISA tests

All plasma samples were diluted fourfold with Bio-Plex sample diluents. Samples were analyzed for three different cytokines (IL-1b, IL-6 and IL-8) by a Bio-Plex Pro™ Human Cytokine Assay (Bio-Rad Laboratories, Inc., Hercules, CA, USA), according to the supplier’s instructions.

### Statistical analysis

Continuous variables are expressed as median (interquartile range), and categorical variables are expressed as frequencies (percentages). Categorical variables were compared by the Chi-Square test or Fisher’s exact test when expected cell frequency was < 5. Continuous variables were compared by the Mann–Whitney U test. The Spearman rank coefficient was calculated for evaluating correlations. For all tests, *P* < 0.05 was considered significant. Graphpad Prism 4.0 (Graphpad Software, La Jolla, CA, USA) was used for statistical analysis.

## Results

### Patients

Twenty septic and 20 control patients were consecutively included in our study. The baseline characteristics of the subjects are presented in Table 1. Most of the septic shock patients had peritonitis or pneumonia, whereas controls had suffered multiple trauma, intracerebral hemorrhage or acute severe bleeding. The SOFA score was significantly higher in the septic group where patients required more norepinephrine and more mechanical ventilation. Except for these parameters, there was no significant difference in baseline characteristics between septic shock cases and controls. In particular, the SAPSII score was not different between the two groups. In order to assess the host response to bacterial exposure, LPS-binding protein (LBP) concentration was measured in plasma. This protein has been used as a potential biomarker reflecting the innate immune response to microbial products. Interestingly, plasma LBP levels were higher in septic versus non-septic cases at day 1 (Table 2). Neutrophil activation by bacterial material may lead to the production of NETs (neutrophil extracellular traps) that can be detected in plasma by measuring cell-free DNA. This marker was also increased under septic conditions at day 1 (Table 2). Finally, patients experiencing a septic shock presented higher cytokine concentrations than control patients, as measured at day 1 (Il-1b = 44.4 pg/ml (20.6–86) versus 16.6 pg/ml (9.9–29), *p* = 0.016; Il-6 = 3363 pg/ml (1069–13,968) versus 317 pg/ml (118–604), *p* < 0.001; Il-8 = 357 pg/ml (187–753) versus 89 pg/ml (62–121), *p* < 0.001) (Additional File 1: Fig. S1).

**Table 1:** Demographic and clinical data of septic shock and non-septic patients

	Septic shock (*n* = 20)	Control (*n* = 20)	*P*
Age [years (*Q*1–*Q*3)]	68 (59.2–76.7)	70.5 (54.2–75.5)	0.65
Male [*n* (%)]	17 (85)	13 (65)	0.14
BMI [kg/m^2^ (*Q*1–*Q*3)]	24.70 (20.2–28.6)	25 (20.2–30.5)	0.65
Dyslipidemia [*n* (%)]	5 (25)	3 (15)	0.69
Statin medication [*n* (%)]	3 (15)	0 (0)	0.23
SAPSII (*Q*1–*Q*3)	39 (29–59.5)	38 (30–51)	0.73
SOFA score (*Q*1–*Q*3)	8 (8–10)	6 (4–8)	< 0.001
Mechanical ventilation [*n* (%)]	20 (100)	14 (70)	0.02
Need for norepinephrine [*n* (%)]	20 (100)	11 (55)	0.001
Norepinephrine (micg/kg/min)	0.42 (0.31–0.64)	0.12 (0.0–0.27)	< 0.001
Enteral nutrition [*n* (%)]	12 (60)	18 (90)	0.065
Parenteral nutrition [*n* (%)]	5 (25)	2 (10)	0.41
Diagnosis at admission [*n* (%)]			
Peritonitis	10 (50)	–	–
Pneumonia	5 (25)	–	–
Cellulitis	2 (10)	–	–
Arthritis	1 (5)	–	–
Hepatic abscess	1 (5)	–	–
Trauma	–	6 (30)	–
Intracerebral hemorrhage	–	6 (30)	–
Traumatic brain injury	–	5 (25)	–
Hemorrhagic shock	–	2 (10)	–

Results are expressed as medians (IQR). Between-group differences were analyzed by a Mann–Whitney test, and a Chi-square test

**Table 2:** Biological data of septic shock and non-septic patients at day 1, day 2 and day 7

	At day 1		At day 2		At day 7	
	Median or % (*Q*1–*Q*3)	*p*	Median or % (*Q*1–*Q*3)	*P*	Median or % (*Q*1–*Q*3)	*p*
Total serum proteins (g/L)						
Non-septic patients	64.5 (55.2–68.7)	0.16	60 (51–62)	0.01	62 (56.5–69.7)	0.06
Septic shock	55 (49.5–65.2)		50.5 (45.5–54.2)		57 (51–52.7)	
Leukocyte/mm^3^						
Non-septic patients	13,460 (8400–15,210)	0.53	11,270 (8630–13,790)	0.09	9070 (6970–12,200)	0.44
Septic shock	13,925 (9000–20,155)		13,945 (10,048–17,133)		10,290 (7910–15,670)	
Neutrophils/mm^3^						
Non-septic patients	10,710 (6318–13,895)	0.84	9640 (8605–11,565)	0.41	7620 (5033–12,833)	0.86
Septic shock	10,120 (6850–18,900)		12,475 (6958–15,788)		7490 (5870–12,425)	
LBP (µg/mL)						
Non-septic patients	39.3 (23.7–47.5)	< 0.001	44.3 (37.8–49.6)	0.09	46.6 (28.9–52.5)	0.45
Septic shock	47.9 (45–49.7)		48.9 (44.4–50.9)		48.8 (43.1–51.3)	
cf-DNA (ng/mL)						
Non-septic patients	582.1 (529.3–694.3)	< 0.0001	730.2 (571.8–806.5)	< 0.0001	866.6 (754.4–958.9)	0.84
Septic shock	980.9 (872.4–1323)		975.1 (841.6–1157)		835.8 (749.3–1023)	

Continuous variables are expressed as medians (interquartile range), and categorical variables are expressed as frequencies (percentages)

A Mann–Whitney test was used for this analysis

Concerning outcome, mortality was 15% (*n* = 3) in the septic group and 10% (*n* = 2) in the non-septic group at day 7 (*P* = 1.0). At day 28, mortality in the septic group patients rose to 25% (*n* = 5) and 20% (*n* = 4) in the non-septic group (*P* = 1.0).

### Lipid profile in septic shock cases and controls

Concentrations of total cholesterol and HDL/LDL-C, but not of triglycerides, were lower in septic shock cases than in controls within 48 h of admission (Table 3). Case–control differences at 24 h were similar to those observed at 48 h for all lipid parameters. The major difference observed between septic and non-septic patients was found for HDL-C that remained significantly different between groups, even at day 7 (whereas septic patients recovered similar LDL-C levels to those of controls). Apolipoprotein A1 concentration was not statistically different at days 2 and 7. Among septic shock cases and controls, we investigated the association of HDL-C with SOFA, SAPSII, LBP and cf-DNA. At day 1, significant negative correlations were observed between HDL-C and cf-DNA (*r* = -0.74, *P *< 0.0001) and LBP (*r* = − 0.31, *P *= 0.004), but not with SOFA and SAPSII scores (all *P* > 0.11). No correlation was observed between HDL concentration and patient outcome (mortality and length of stay in ICU) in either septic or control patients.

**Table 3:** Lipid parameters in septic patients and controls

Lipid profile at day 1	Septic shock (*n* = 20)	Non-septic patients (*n* = 20)	*p*
Total cholesterol, mmol/l	2.1 [1.6–2.7]	4.0 [3.1–4.8]	< 0.0001
mg/dl	82 [62–105]	156 [121–187]	
LDL-C, mmol/l	1.0 [0.8–1.6]	2.3 [1.4–2.9]	0.001
mg/dl	39 [31–62]	89 [54–112]	
HDL-C (mmol/l)	0.4 [0.3–0.7]	1.3 [1.0–1.5]	<0.0001
mg/dl	15 [12–27]	50 [39–58]	
Triglycerides, mmol/l	0.9 [0.7–1.7]	1.0 [0.7–1.2]	0.69
mg/dl	80 [62–150]	88 [62–106]	
Apolipoprotein-A1, mg/l	916 [315–2543]	2482 [1428–5518]	0.05

Lipid profile at day 2	Septic shock (*n* = 18)	Non-septic patients (*n* = 19)	*p*
Total cholesterol, mmol/l	1.8 [1.2–2.9]	3.2 [2.7–3.9]	0.001
mg/dl	70 [47–113]	125 [105–152]	
LDL-C, mmol/l	0.9 [0.4–1.5]	1.5 [1.1–2.2]	0.02
mg/dl	35 [15–58]	58 [43–86]	
HDL-C, mmol/l	0.3 [0.2–0.6]	1.0 [0.9–1.4]	<0.0001
mg/dl	12 [8–24]	39 [35–54]	
Triglycerides, mmol/l	1.4 [0.9–1.7]	1.0 [0.8–1.1]	0.16
mg/dl	124 [80–150]	88 [71–97]	
Apolipoprotein-A1, mg/l	787 [357–2234]	1562 [965–3101]	0.22

Lipid profile at day 7	Septic shock (*n* = 15)	Non–septic patients (*n* = 12)	*p*
Total cholesterol, mmol/l	2.7 [1.7–3.5]	3.4 [2.2–3.8]	0.29
mg/dl	105 [66–136]	132 [86–148]	
LDL-C, mmol/l	1.5 [0.9–2.0]	1.8 [1.4–2.5]	0.38
mg/dl	58 [35–77]	70 [54–97]	
HDL-C, mmol/l	0.4 [0.3–0.6]	0.7 [0.5–1.2]	0.04
mg/dl	15 [12–24]	27 [19–46]	
Triglycerides, mmol/l	1.3 [1.1–1.6]	1.0 [0.9–1.5]	0.25
mg/dl	115 [97–142]	88 [80–133]	
Apolipoprotein-A1, mg/l	1340 [391–1922]	962 [508–3752]	0.98

Results are expressed as medians (IQR). The statistical differences were analyzed by a Mann–Whitney test

### Lipoprint analysis

**Fig. 1:**
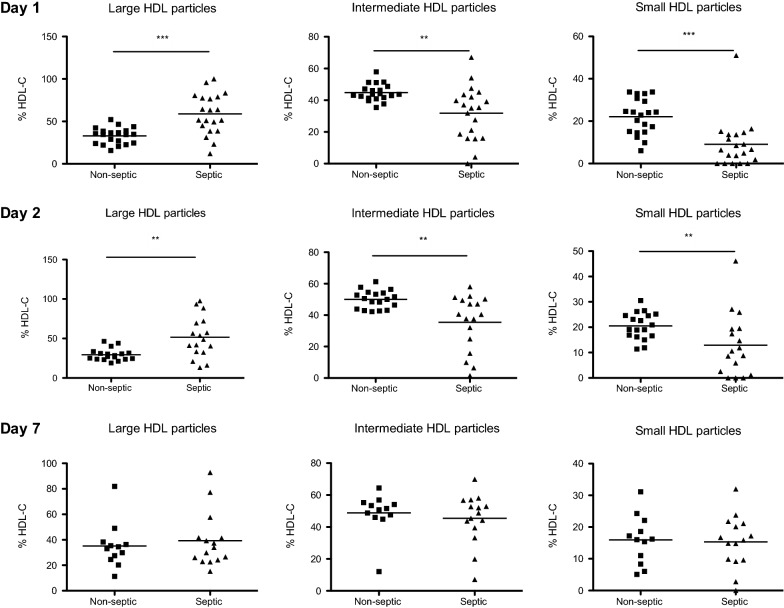
Lipoprint analysis of HDL particle size. Distribution of the different HDL subfractions (large, intermediate and small) in plasma of septic patients and non-septic patients at day 1, day 2 and day 7. The results are presented as percentages of total cholesterol detected in HDL fractions. *p < 0.05, **p < 0.01, ***p < 0.001 (Mann–Whitney)

A major change in the distribution of HDL particles was observed in septic versus non-septic patients. A statistically significant decrease in small and intermediary fractions, in parallel with an increase in large particles, was observed at 24 h and at 48 h in septic versus non-septic patients (Fig. 1). At day 7, septic patients presented similar HDL profiles to those of non-septic subjects (Fig. 1). Thus, in addition to decreased levels of HDL-C, the remaining HDL particles were larger in septic versus non-septic patients. While we described a significant difference in HDL size between septic and non-septic patients, we did not find any correlation between HDL size at day 1, 2 or 7 and patient outcome (mortality or length of stay in ICU) in either septic or control patients. Interestingly, there was a significant positive correlation between the percentage of large particles at day 1 and LBP concentration (*r* = 0.39, *p* = 0.012) and a significant negative correlation between percentage of small particles at day 1 and LBP concentration (*r* = − 0.5, *p* = 0.001) (Additional File 2: Fig. S2). No such correlation between particles size and LBP concentration was found at day 2 and 7.

## Discussion

In the present study, we have compared a group of septic shock patients to a control group consisting of patients with a systemic inflammatory response syndrome having suffered traumatic brain injury or multiple traumas, intracerebral hemorrhage or acute severe bleeding. These two groups are comparable in terms of SAPSII. We have shown that at admission to ICU, HDL-C concentrations were lower and HDL particles were larger in septic versus non-septic patients. This point is of interest since small HDLs are usually considered as functional particles; in sepsis, there is not only a shift toward large non-functional HDLs but also a marked decrease in the concentration of these lipoproteins (HDL cholesterol).

We show that LBP, an acute phase protein predominantly synthesized by the liver in response to gram-negative bacteria [26], is significantly higher in septic versus non-septic patients at 24 and 48 h after admission. The higher cell-free DNA concentration in plasma attested the increased cell death in septic conditions. The release of extracellular DNA is a well-known active phenomenon for neutrophils that are over-stimulated (producing Neutrophil Extracellular Traps, NETs), in particular with LPS [25, 27]. In our conditions, it cannot be excluded that cells other than neutrophils may contribute to the increased cf-DNA concentration in septic patients. Neutrophils isolated from septic patients have been reported to be less prone to release DNA than those from non-septic subjects; this was paralleled by an increased NET concentration in the septic group [28], suggesting that these cells have already been activated and have subsequently released cf-DNA in plasma.

### Lipid profile: HDL particle size in septic versus non-septic and septic patients

By comparing septic shock patients to control ICU patients of similar severity, we show that the lipid profile is profoundly modified under septic conditions. Whereas both LDL-C and HDL-C were lower in septic patients, triglyceride levels were not significantly different between the two groups, albeit slightly higher in septic patients at days 2 and 7. Numerous studies have reported that the serum lipid profile is modified in septic relative to non-septic patients (for review, see [29]). These changes were documented more than 30 years ago [30]. In our study, it is unlikely that lipid changes are due to beta-blockers or angiotensin converting enzyme inhibitors since these drugs do not significantly modify HDL levels. Also, no differences in these treatments were observed between groups (data not shown). However, in a recent work by Khera et al., a 12-month rosuvastatin treatment (20 mg/day) did not change the cholesterol efflux capacity of HDLs, but led to an increase in HDL cholesterol (+ 7.7%), apoA-I (+ 4.3%), and HDL particle number (+ 5.2%) [31]. In our cohort of patients, no differences were observed regarding statin medication between the two groups.

To our knowledge, this is the first demonstration that septic shock patients present a shift of HDL size from small to large particles compared to non-septic patients. In accordance with our results, De la Llera-Moya et al. [32] reported that LPS-induced endotoxemia in healthy volunteers led to a depletion of pre-beta1a and small- and medium-sized HDL particles, determined by 2D eletrophoresis and nuclear magnetic resonance. Interestingly, small HDL particles were shown to be more antioxidant and more anti-inflammatory in cardiovascular disease [33], but large HDL particles as determined by Lipoprint technology appear to be more associated with a reduced risk of coronary artery disease [17, 18].

### HDLs: therapeutic option or surrogate marker?

Globally, HDL particles exert a variety of protective effects on endothelial cells [33] that may modulate the physiological response under inflammatory conditions [34]. For example, HDLs have been shown to modulate vascular cell activation [13, 35] and endothelial expression of adhesion molecules [36] that may in turn reduce leukocyte extravasation. In addition, HDLs have been reported to interact with LPS and to facilitate its clearance by different organs [37]. However, different therapeutic strategies aiming at inhibiting LPS binding to TLR4 failed to improve the outcome of severe sepsis patients (no reduction in 28-day and 1-year all-cause mortality) [38]. HDLs, in addition to their LPS-scavenging capacity, may provide a more global endothelial protection and leukocyte pacification that may account for their protective role under inflammatory conditions. Barlage et al. suggested that the beneficial effects of HDLs on gram-positive infections may be due to other anti-inflammatory properties, including the modulation of neutrophil activation [22]. HDLs could also provide a protective effect in sepsis due to their antioxidant capacity. The infusion of reconstituted HDLs (that are considered to be small HDL particles) has been shown to modulate inflammatory parameters in different animal or human settings of endotoxemia [36, 39, 40]. Specifically in rodent models of sepsis, infusion of reconstituted HDLs or mimetic peptides improved survival [36, 41–43].

Moreover, because sepsis still remains an important cause of mortality and morbidity, early biomarkers could be useful to establish a diagnosis of sepsis and also to indicate its severity. To date, there is no biomarker that fulfills these objectives in terms of sensitivity and specificity [4]. The use of HDL concentration, size and/or functionality may thus represent an early and sensitive biomarker of sepsis, low levels being associated with severity and poor outcome. Interestingly, a recent study has shown that variations in genes involved in HDL metabolism could contribute to changes in HDL-C level; a rare missense variant in CETP (rs1800777-A) was thus associated with significant reductions in HDL-C concentration during sepsis [44]. Carriers of the A allele had an increased mortality and morbidity compared to non-carriers.

### Some hypotheses concerning the mechanistic basis of our findings

- First, the interaction between HDL particles and bacterial components (LPS or LTA) could produce changes in HDL size and potential functions.
- Second, septic conditions and especially a procoagulant state could lead to the aggregation of HDL particles that could participate in increasing HDL size according to the Lipoprint technology.
- Third, sepsis impairs the capacity of HDL to induce cellular cholesterol efflux. ABCA-1 dysfunction could lead a decrease in the synthesis of small pre-β HDL. Moreover, the SRB-1 depletion observed in case of inflammatory condition such as sepsis could also participate in increasing the proportion of large HDL-2 and HDL-3 proportion.
- Lastly, the septic condition increases HDL-associated Serum Amyloid A (SAA). HDL size could be impacted by this association.

### Our study has several limitations

- First, this is a monocentric study with a small population size. However, with only 20 patients per group, we have found a significant difference in HDL particle size between the two groups.
- Second, our analysis was performed using only one electrophoresis system (lipoprint). Other techniques such as nuclear magnetic resonance should also be performed.
- Third, more patients received norepinephrine in septic versus the control group. Norepinephrine stimulates the expression of lipoprotein lipase, which exerts an important role in the metabolism of lipids by hydrolyzing triglycerides contained in chylomicrons and VLDL. Its overexpression may participate in lipid profile modifications under septic conditions.
- Lastly, we did not show in this study any mechanistic pathway explaining the shift toward the large size.

## Conclusion

Our study confirms that HDL-C concentration is very low in septic relative to non-septic patients admitted to our ICU and demonstrates for the first time that HDL particles are larger under septic conditions. Further studies should be conducted in order to understand the mechanisms of this shift. According to numerous experimental studies [36, 42, 43, 45, 46], HDL supplementation may represent a potential therapy for septic patients. However, additional evidence should be provided in animal models, particularly on hard endpoints such as mortality in order to translate HDL therapy into clinical practice.

## Additional files

**Additional file 1: Figure S1.** Cytokine concentrations at day 1. Il-1b, Il-6 and Il-8 concentrations between septic and non-septic patients at day 1. *: *p* < 0.05, ** *p* < 0.01, ***: *p* < 0.001 (Mann–Whitney).

**Additional file 2: Figure S2.** Correlation between the percentage of large and small HDL particles and LBP concentration at day 1.

## Data Availability

The datasets used and analyzed during the current study are available from the corresponding author on reasonable request.

## Abbreviations

AIDS: acquired immune deficiency syndrome
HDL: high-density lipoprotein
ICU: intensive care unit
IL-1b: interleukin 1b
IL-6: interleukin 6
IL-8: interleukin 8
LBP: lipopolysaccharide
LDL: low-density lipoprotein
NET: neutrophil extracellular traps
NO: nitric oxide
PAF-AH: platelet-activating factor acetylhydrolase
PON1: paraoxonase
TLR-4: toll-like receptor 4
TNF-alpha: tumor necrosis factor-alpha
SAPS II: simplified acute physiology score II
SAA: serum A amyloid
SOFA: sequential organ failure assessment
