# Supplementary material for: High-density lipoprotein (HDL) particle size and concentration changes in septic shock patients
Source: Ann Intensive Care. 2019 Jun 13;9:68. doi: 10.1186/s13613-019-0541-8 (PMC6565796; doi:10.1186/s13613-019-0541-8)
Supplement: Supplementary file 1 — Additional file 1: Figure S1. Cytokine concentrations at day 1. Il-1b, Il-6 and Il-8 concentrations between septic and non-septic patients at day 1. *: p < 0.05, ** p < 0.01, ***: p < 0.001 (Mann–Whitney). [file 13613_2019_541_MOESM1_ESM.docx]

Figure S1: Cytokine concentrations at day 1 (*: p<0.05; ***: p<0.001)
