# Supplementary material for: High-density lipoprotein (HDL) particle size and concentration changes in septic shock patients
Source: Ann Intensive Care. 2019 Jun 13;9:68. doi: 10.1186/s13613-019-0541-8 (PMC6565796; doi:10.1186/s13613-019-0541-8)
Supplement: Supplementary file 2 — Additional file 2: Figure S2. Correlation between the percentage of large and small HDL particles and LBP concentration at day 1. [file 13613_2019_541_MOESM2_ESM.docx]

Figure S2: correlation between the percentage of large and small HDL particles and LBP concentration at day 1
